# Foretinib Is Effective against Triple-Negative Breast Cancer Cells MDA-MB-231 In Vitro and In Vivo by Down-Regulating p-MET/HGF Signaling

**DOI:** 10.3390/ijms24010757

**Published:** 2023-01-01

**Authors:** Xiwei Ji, Xiangrui Meng, Qingfeng He, Xiaoqiang Xiang, Yufei Shi, Xiao Zhu

**Affiliations:** 1 Institute of Clinical Pharmacology, Peking University First Hospital, Beijing 100034, China; 2 Intensive Care Unit, Xiyuan Hospital of China Academy of Traditional Chinese Medicine, Beijing 100091, China; 3 Department of Clinical Pharmacy and Pharmacy Administration, School of Pharmacy, Fudan University, Shanghai 201203, China

**Keywords:** foretinib, MDA-MB-231, triple-negative breast cancer, p-MET, HGF, pharmacokinetics

## Abstract

This study investigated the antitumor effects of foretinib on triple-negative breast cancer cells MDA-MB-231 xenograft tumors in vivo underlying phosphorylated mesenchymal to epithelial transition (p-MET)/ hepatocyte growth factor (HGF)-related mechanism, as well as its pharmacokinetic characteristics. The MDA-MB-231 human breast cancer cell line was used for in vitro experiments, and the tumor xenograft model was established for in vivo experiments. MDA-MB-231 xenograft mice received oral foretinib (15 or 50 mg/kg/day) or vehicle for 18 days. The xenograft tumors were collected. Protein expressions of p-MET and HGF were examined with Western blotting and immunohistochemical staining. The mRNA expression of *MET* was examined with real-time PCR. Blood samples were collected from the mice treated with foretinib under different doses of 2, 10, and 50 mg/kg, and the pharmacokinetic profiles of foretinib were evaluated. We found that foretinib treatment caused a significant inhibition in tumor growth in a dose-dependent manner, whereas the continuous administration did not result in weight loss in treated nude mice. In both MDA-MB-231 cells and xenograft tumors, foretinib suppressed the expression of p-MET and HGF. These findings reveal that the decrease of p-MET and HGF may play an important role in the anti-breast cancer properties of foretinib.

## 1. Introduction

Triple-negative breast cancer (TNBC) is a subtype with a poor prognosis and overall survival. It does not express estrogen receptor α, progesterone receptor, and human epidermal growth factor receptor 2 (HER2). Therefore, TNBC lacks molecular targets for effective therapeutics [1,2].

Mesenchymal to epithelial transition (MET) is a receptor tyrosine kinase (RTK), which is overexpressed in 20–30% of breast cancer cases [3]. MET overexpression promotes the growth, survival, and migration of cancer cells and appears to be associated with a worse prognosis and a metastatic phenotype. MET can be activated through mechanisms, including ligand-dependent and ligand-independent activation, such as mutation autocrine or paracrine stimulation [4,5]. The ligand-dependent activation of MET occurs in the mammary gland, where stromal cells produce hepatocyte growth factor (HGF) [6]. When bound to the ligand (i.e., HGF), the kinase domain is activated and generates phosphorylated MET (p-MET), which activates a number of downstream signaling routes, such as rat sarcoma viral oncogene homolog (RAS) and extracellular signal-related kinase (ERK) [7,8]. The abnormal HGF/MET signaling pathway confers an aggressive phenotype with a high risk of progression and poor outcome [9,10].

Foretinib is a multitargeted small-molecule kinase inhibitor that targets HGF and vascular endothelial growth factor (VEGF) receptor tyrosine kinases, platelet-derived growth factor receptor β (PDGF-Rβ), Tie-2, FMS-like tyrosine kinase-3 (FLT-3), AXL, 3-KIT, and RON [11]. As a potent inhibitor of MET, foretinib suppressed different MET-activated cell lines and reduced tumor growth in different animal studies [12,13]. Foretinib inhibited the HGF-induced MET phosphorylation and prevented the HGF-induced responses of tumor cells. It is believed that p-MET is a promising therapeutic target due to its elevated expression in multiple TNBC subtypes, which indicates that, by inhibiting the HGF/MET signaling pathway, foretinib may exert an antitumor effect through a direct cytotoxic effect on tumor cell proliferation [14,15].

This study aims to investigate the inhibition of foretinib on TNBC in vitro and in vivo and to achieve a better understanding of the potential role of p-MET inhibition in TNBC treatment using foretinib. To accomplish this, we examined the antitumor effects of foretinib in MDA-MB-231 xenograft tumors in vivo and then investigated the effects of foretinib on p-MET/HGF signaling in both breast cancer cells and xenograft tumors.

## 2. Results

### 2.1. Foretinib Inhibited the Tumor Growth of MDA-MB-231 Xenografts

**Figure 1:**
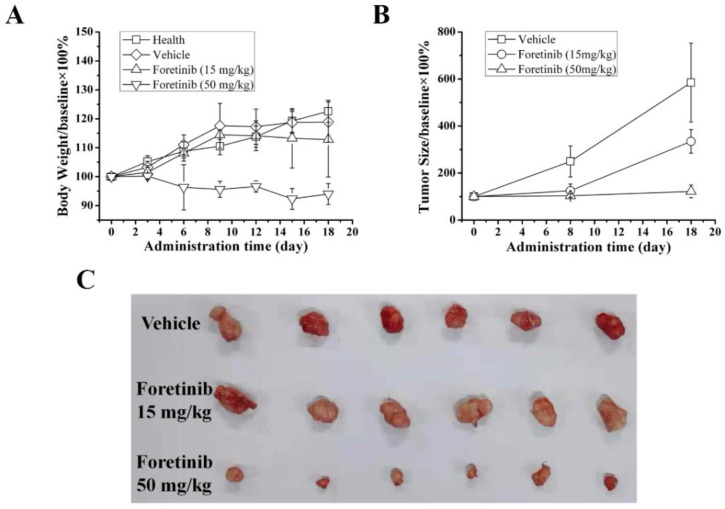
Inhibitory effects of foretinib on MDA-MB-231 xenograft tumors. (**A**) Body weights at different timings in the foretinib-treated groups were similar to those in the vehicle-treated group, indicating that foretinib has low toxicity. (**B**) Foretinib treatment caused a significant inhibition in tumor growth compared with vehicle treatment. (**C**) Photos of the removed tumors of different groups. Each group contains six mice (*n* = 6).

Foretinib can decrease the cell viability of MDA-MB-231, as we have previously described [16]. Compared to the vehicle group, foretinib exerts significant inhibitory effects on MDA-MB-231 xenograft tumor growth in a dose-dependent manner (Figure 1C). On day 18, the tumor inhibitory rates of foretinib at the dosages of 15 and 50 mg/kg were 42.79 ± 8.52% and 79.16 ± 4.58%, respectively (Figure 1B). The mean tumor volume of the high dose group is 80.67 ± 17.00 mm^3^, which is significantly smaller than those of both the vehicle group (285.00 ± 30.82 mm^3^) and the low dose group (220.00 ± 70.14 mm^3^). The body weights of low dosage (15 mg/kg) foretinib-treated mice in different groups were similar to those of the vehicle-treated mice (Figure 1A). These results suggest that foretinib inhibits the growth of MDA-MB-231 xenograft tumors and has low toxicity.

### 2.2. Pharmacokinetic Characteristics of Foretinib In Vivo

**Figure 2:**
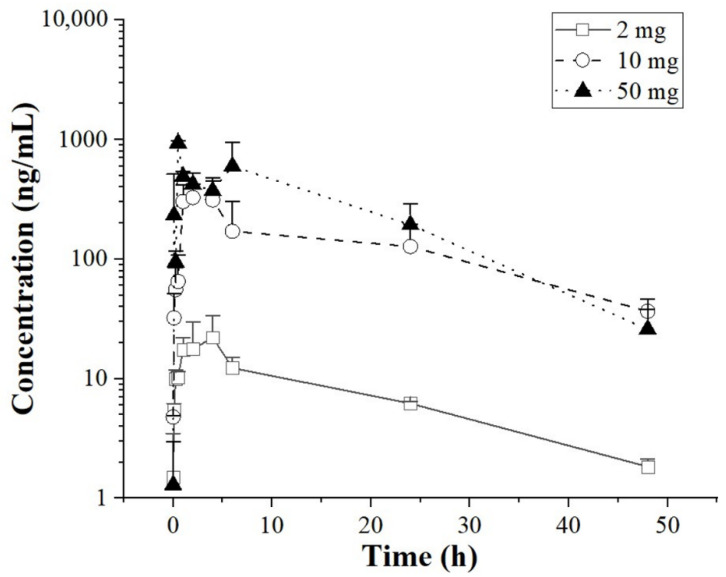
The pharmacokinetic characteristics of foretinib in tumor-bearing mice. The squares, triangles, and circles represent the plasma concentrations of foretinib over time after intragastric administration in the tumor-bearing mice at the dosages of 2, 10, and 50 mg/kg, respectively.

The plasma concentration-time profiles of foretinib after the single oral doses of 2, 10, and 50 mg/kg are illustrated in Figure 2, and the main estimated pharmacokinetic parameters are listed in Table 1. The plasma concentration of foretinib reduced to a low level at 48 h after administration. The half-life (T_1/2_) of different dose groups were 15.09 ± 2.49, 19.95 ± 6.72, and 9.48 ± 0.045 h, respectively, which suggested that foretinib was eliminated rapidly in vivo after oral administration.

### 2.3. Foretinib Significantly Inhibited p-MET In Vitro and In Vivo

**Figure 3:**
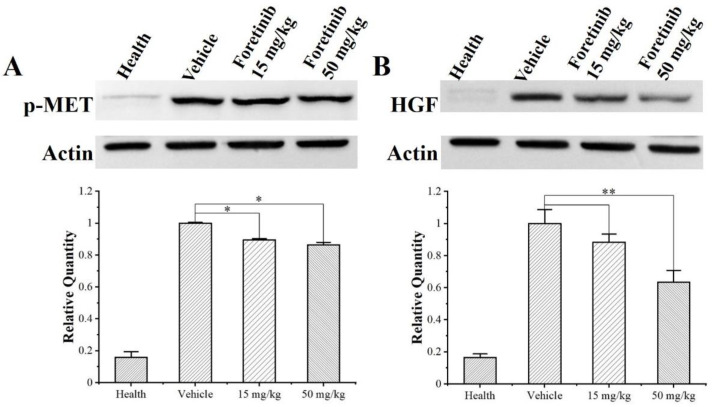
Foretinib inhibited p-MET and HGF in breast tissues of healthy mice and MDA-MB-231 xenograft tumor as revealed by Western blot analysis. Tumor-bearing mice were treated with foretinib (15 and 50 mg/kg) and vehicle. (**A**) Foretinib reduced the expression of p-MET in tumor tissues. (**B**) Foretinib reduced the expression of HGF in MDA-MB-231 tumor tissues. The expression of β-actin was included as a loading control for both p-MET and HGF. Each bar corresponds to the mean ± SD of three independent experiments (*n* = 3). *, *p* < 0.05; **, *p* < 0.01, vehicle treatment group.

**Figure 4:**
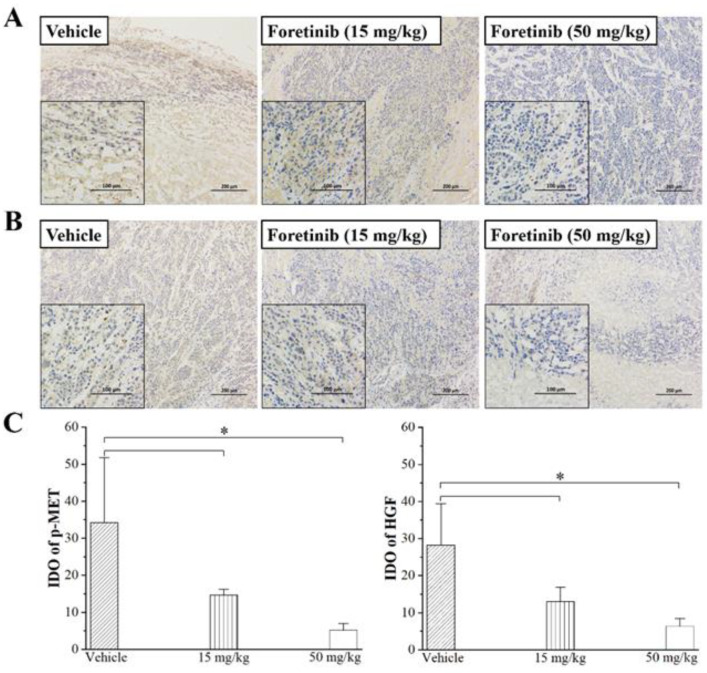
Immunostaining on tumor paraffin sections from mice treated with vehicle and foretinib (15 and 50 mg/kg) for 18 days demonstrated the reduction of p-MET (**A**) and HGF (**B**) by foretinib. The brown staining intensity represents the expression of p-MET and HGF in the cytosol of tumors, and the blue staining represents the cell nucleus. The original magnification is 100× and 200× for all panels. (**C**) Quantitative analysis of IODs of anti-p-MET (left) and anti-HGF (right) immunohistochemical staining at the different dosages. Magnification, 200×. *, *p* < 0.05. IOD, integrated optical density.

Western blot analysis showed that the expressions of p-MET and HGF are very low in normal breast tissues but are excessive in tumor tissues of the MDA-MB-231 tumor-bearing nude mice. Treatment with foretinib can significantly inhibit the expression of p-MET and HGF in the tumor. The max inhibitory ratios are 13.6% and 36.5%, respectively (Figure 3). Immunohistochemical (IHC) staining and integrated optical density (IOD) also indicated that p-MET and HGF expression in the tumor tissues of the mice decreased in a dose-dependent manner after treatment with foretinib (Figure 4); p-MET and HGF protein expressions are represented by the brown staining.

**Figure 5:**
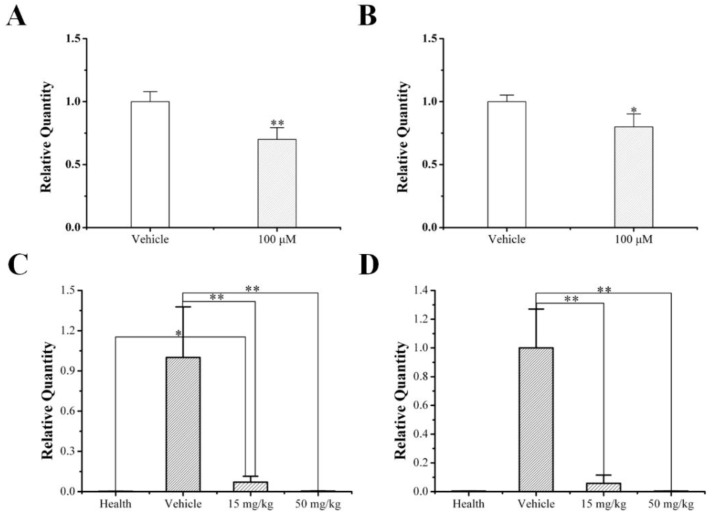
Foretinib reduced the mRNA expression of *MET* (**A**,**C**) and *HGF* (**B**,**D**) in MDA-MB-231 cells and xenograft tumors. The expression of *GAPDH* was included as a loading control. *, *p* < 0.05; **, *p* < 0.01, compared with the vehicle control.

Consistent with the Western blot and IHC results, foretinib effectively suppressed the mRNA expression levels of *MET* and *HGF* in vitro and in tumors (Figure 5). Real-time PCR results suggested that the regulation of *MET* and *HGF* expression by foretinib is mainly at the gene transcriptional level.

## 3. Discussion

The results of the tumor growth inhibition assay indicated that the high-dose foretinib treatment (50 mg/kg/day) caused a remarkable suppression in tumor growth compared with those of the low-dose group (15 mg/kg/day) and vehicle-treated controls. In a previous study, we investigated the inhibitory effects of foretinib on MDA-MB-231 cells [16]. The in vitro results indicated that foretinib could arrest cancer cells mainly in the G2/M phase and induce marked apoptosis, which may contribute to the antitumor activity and cytotoxic effectivity of foretinib.

A previous study indicated that foretinib could remarkably inhibit the expression of p-MET in MDA-MB-231, but the expression of MET remained unchanged [17]. We also demonstrated that foretinib could inhibit the expression of p-MET in MDA-MB-231 xenograft tumors. A Western blot analysis showed that p-MET and HGF protein levels in the vehicle treatment group were significantly higher than those in breast tissues of healthy nude mice, and treatment with foretinib significantly decreased the level of upstream p-MET protein. Our IHC staining results also confirmed these findings. Compared with the vehicle treatment group, the mRNA expression of *MET* and *HGF* in tumors was significantly inhibited by the administration of foretinib in a dose-dependent manner, which was consistent with the results of Western blot and IHC staining. The observed net *MET* and *HGF* mRNA levels are the sum of reduction by foretinib and induction by natural cancer disease progression.

It was reported that TNBC cells exhibit the perpetual phosphorylation of EGFR, even in the presence of tyrosine kinase inhibitors (TKI), which correlates with the resistance to these inhibitors induced by MET–EGFR crosstalk. EGFR and MET may interplay independently of both receptor activities, which can promote resistance to EGFR inhibitors via the constitutive phosphorylation of MET [18,19]. In this scenario, the combinations of MET and EGFR inhibitors may provide a potential therapeutic strategy for TNBC treatment [5]. Furthermore, our previous study suggested that foretinib can cause cancer cells to accumulate in the G2/M phase and thus inhibit the metastasis of cells [16]. Therefore, the effects on cell cycles should be considered in combinations of foretinib and other antitumor drugs. One aim of our study is to support this combination regimen.

The main limitation of this study was the small sample size of mice in the pharmacokinetic study. Only the blood samples of three mice at each time point were collected to determine the plasma concentrations of foretinib, although the variations among individuals are minor.

In summary, this is a study of the anti-TNBC effects of foretinib and its mechanism of action. Our studies demonstrate that inhibiting the autophosphorylation of p-MET plays an important role in the anti-TNBC effect of foretinib. Furthermore, we investigated the pharmacokinetic characteristics of foretinib in the tumor xenograft model, which could help describe the quantitative relationship between the drug concentration and tumor size. With its ideal pharmacokinetic characteristics, low toxicity, and p-MET/HGF pathway inhibition, foretinib displays promising chemotherapeutics for treating and preventing TNBC.

## 4. Materials and Methods

### 4.1. Drugs and Reagents

Foretinib (>99%) was purchased from Selleck (Houston, TX, USA). Dulbecco’s Modified Eagle Medium (DMEM), the media were purchased from Thermo Co., Ltd. (Waltham, MA, USA), and fetal bovine serum (FBS) was purchased from Hyclone (Logan, UT, USA). Primary antibodies against p-MET, HGF, glyceraldehyde-3-phosphate dehydrogenase (GADPH), and β-Actin and horseradish peroxidase-conjugated anti-mouse secondary antibodies were obtained from Abcam (Cambridge, UK).

### 4.2. Cell Culture

The MDA-MB-231 cell line was obtained from the cell bank of the Cancer Institute & Hospital, Chinese Academy of Medical Science. MDA-MB-231 cells were cultured in DMEM medium, which was supplemented with 10% FBS, 100 U/mL penicillin, and 100 μg/mL streptomycin. The cells were maintained at 37 °C in a mixture of 5% CO_2_ atmosphere.

### 4.3. Animals

Beijing Vital Laboratory Animal Technology (Beijing, China) provided female BALB/c nude mice aged six weeks (Certificate No. SCXK 2019-0010). The animal procedures were approved by the Department of Laboratory Animal Science of Peking University Health Science Center (Beijing, China, No. LA2018018). These nude mice were housed under standard temperature (25–28 °C), humidity (50–60%), and light (12 h light/12 h dark) conditions with free access to food and water before being used for the study.

### 4.4. Tumor Xenograft Model

MDA-MB-231 cells (2 × 10^7^) were suspended in 200 μL of PBS (pH 7.4) and were inoculated subcutaneously into the second mammary fat pads of the nude mice. The tumor diameter was measured with a vernier caliper and converted into tumor volume using the formula 1/2 × A × B^2^ (A = larger diameter, B = smaller diameter). The treatment was started when the average tumor volume reached 50 mm^3^.

### 4.5. Tumor Growth Inhibition Assay

Xenograft mice were randomly divided into three groups, with six in each group. Foretinib was dissolved in a 15% hydroxypropyl-β-cyclodextrin aqueous solution and administered by intragastric gavage of 15 or 50 mg/kg/day. The blank control group received only the vehicle solution. The tumor size and body weight were measured every three days. After 18 days of treatment, the animals were euthanized by cervical displacement. The tumors were collected after the final treatment and frozen at −80 °C until use.

### 4.6. Pharmacokinetic Study

A liquid chromatography-tandem mass spectrometry (LC-MS/MS) method was developed by our group to determine the concentrations of foretinib in plasma [20]. According to the study, tumor-bearing nude mice received an intragastric administration of foretinib at 2, 10, and 50 mg/kg, and the blood samples were obtained at 0, 0.083, 0.25, 0.5, 1, 2, 4, 6, 24, and 48 h with three mice at each time point. The serum concentrations of foretinib were detected by the LC-MS/MS method established previously. Data processing was performed with Phoenix WinNonlin 8.1.0.3530 (Pharsight Corporation, Mountain View, CA, USA).

### 4.7. Western Blot Analysis

*In vivo,* tumor-bearing mice were treated with foretinib at 15 or 50 mg/kg/day for 18 days. On day 19, the tumors were collected and then homogenized in 50 mmol/L Tris buffer (pH 7.4) containing 0.25 mol/L sucrose, 3 mmol/L β-mercaptoethanol, and 0.02% (*v*/*v*) Tween-20. All homogenates were centrifuged at 40,000× *g* for 1 h at 4 °C and the protein concentrations of cytosol were determined using the BCA protein assay kit above. Cytosol aliquots were collected and stored at −80 °C. For each treatment group, the cytosol samples with the same total protein of each tumor were mixed for Western blot analysis.

Total protein (50 μg) was separated by SDS-12% *w*/*v* polyacrylamide gel electrophoresis and transferred to PVDF membranes (Pierce, Rockford, IL, USA). The antibodies used were as follows: p-EGFR, EGFR (1:1500); p-ERK, ERK (1:2000). Horseradish peroxidase-linked anti-mouse antibody (1:6000) was used as a secondary antibody. The membrane was developed by Supersignal Ultra (Pierce, Rockford, IL, USA). The densitometric quantification of protein bands was obtained using ChemiDoc XRS+ System (Bio-Rad, Hercules, CA, USA). Three animals were used in each group of the treatment and each experiment was repeated three times.

### 4.8. Quantitative Real-Time PCR

In vitro, MDA-MB-231 cells were prepared as described above. *In vivo*, the MDA-MB-231 xenograft mice were randomly divided into two groups and treated with vehicle solution and 150 mg/kg foretinib, respectively. Tumors were obtained from three mice per group at 0, 3, 6, 9, and 12 days. The total RNA was extracted using an RNA isolation kit (Takara, Shiga, Japan) according to the manufacturer’s instructions. cDNA was synthesized from 2 μg of RNA using a reverse transcriptase M-MLV synthesis kit (Takara). Real-time PCR was performed using the MyiQ5 real-time PCR detection system (Bio-Rad) with SYBR Premix Ex Taq (Takara). The PCR products for human β-actin, *p-MET,* and *HGF* were synthesized with the primer pairs FP (forward primer) 5′-TGCTGAGTATGTCGTGGAG-3′ and RP (reverse primer) 5′-GTCTTCTGAGTGGCAGTGAT-3′ (β-actin, Gene ID: 60), FP 5′-AGCGTCAACAGAGGGACCT-3′ and RP 5′-GCAGTGAACCTCCGACTGTATG-3′ (*MET*, Gene ID: 4233), as well as FP 5′-CAGTCAGCACCATCAAGGCAAGG-3′ and RP 5′-GCACATCCACGACCAGGAACAAT-3′ (*HGF*, Gene ID: 3082). All primers were purchased from AuGCT DNA-SYN Biotechnology Co., Ltd. (Dalian, China). Samples were run in triplicate under the following conditions: an initial denaturation for 30 s at 95 °C, followed by 45 cycles of 15 s at 95 °C, 30 s at 60 °C, and 33 s at 72 °C. The levels of gene expression in each sample were normalized to β-actin mRNA.

### 4.9. Immunohistochemical (IHC) Staining

After the paraffin sections of tumors were deparaffinized, rehydrated, and washed with PBS (pH 7.4), antigen retrieval was performed by boiling the sections in 10 mmol/L sodium citrate buffer (pH 6.0) in the microwave for 4 min. The endogenous peroxidase activity was quenched by placing the slides in 3% hydrogen peroxide in methanol. The slides were then blocked with 3% BSA-PBS, followed by incubation with a 1:425 dilution of the primary antibody of p-MET and HGF (Cell Signaling Technology, Danvers, MA, USA) overnight at 4 °C. Thereafter, the detection was performed using a biotin-conjugated secondary antibody and a streptavidin-horseradish peroxidase conjugate (SA-HRP), followed by colorimetric detection using diaminobenzidine (DAB). The sections were counterstained with hematoxylin and stabilized with a mounting medium. The immunohistochemical images with integrated optical density (IOD) of positive expression, which reflected the distribution characteristics of anti-p-MET and anti-HGF positive stained area in tumor tissues, were quantitatively analyzed using Image-Pro Plus version 6.0 (Media Cybernetics, Inc., Silver Spring, MD, USA).

### 4.10. Statistics

When applicable, results were presented as the mean ± SD. One-way analysis of variance (ANOVA) was used to determine the significance among groups, after which *post hoc* tests with the Bonferroni’s correction were used for multiple comparisons among individual groups. Differences were considered statistically significant at *p* < 0.05. Statistical analyses were performed using GraphPad Prism 8.0 software (GraphPad software, Inc., San Diego, CA, USA).

## Figures and Tables

**Table 1:** Parameter estimates obtained from the pharmacokinetic analysis.

Parameters *	Foretinib		
Dose (mg/kg)	2	10	50
T_1/2_ (h)	15.09 ± 2.49	19.95 ± 6.72	9.48 ± 0.045
T_max_ (h)	4	2	0.5
C_max_ (ng/mL)	22.25 ± 11.24	334.00 ± 107.48	1434.00 ± 743.88
k (1/h)	0.046 ± 0.008	0.037 ± 0.012	0.073 ± 0.0003
*V*_d_ (L/kg)	109.72 ± 34.73	39.76 ± 13.95	60.90 ± 31.53
AUC_0–t_ (μg·h/L)	366.29 ± 76.42	6207.91 ± 423.10	12,633.22 ± 6499.58
AUC_0–∞_ (μg·h/L)	406.88 ± 63.35	7257.16 ± 108.13	12,988.54 ± 6777.58
MRT (h)	20.13 ± 5.05	25.06 ± 8.36	13.31 ± 0.73

* T_1/2_, half-life; T_max_, time to reach maximum concentration; C_max_, maximum concentration; k, elimination constant; *V*_d_, volume of distribution; AUC_0–t_, area under the curve up to the last measurable concentration; AUC_0–∞_, area under the curve to infinite time; MRT, mean resident time.

## Data Availability

The data will be available from the corresponding author following reasonable request.
